# ﻿Redescription of two species of *Microcyclops* (Copepoda, Cyclopoida) and use of ordination models to classify American species

**DOI:** 10.3897/zookeys.1173.97827

**Published:** 2023-08-03

**Authors:** Martha Angélica Gutiérrez-Aguirre, Adrián Cervantes-Martínez

**Affiliations:** 1 Universidad Autónoma del Estado de Quintana Roo (UQROO), Campus Cozumel, Av. Andrés Quintana Roo s/n, 77600, Cozumel, Quintana Roo, Mexico Universidad Autónoma del Estado de Quintana Roo Cozumel Mexico

**Keywords:** Classification, diversity, freshwater, species richness, taxonomy

## Abstract

Two species of the freshwater copepod genus *Microcyclops* are redescribed, *M.finitimus* Dussart, 1984, and *M.minor* Dussart, 1984 from type specimens. Redescription includes the microstructure of intercoxal sclerites and the basipodites of thoracic appendages, as well as the urosomal microstructure. According to the cluster (UPGMA and Euclidean distance) and PCA analyses performed, it was possible to improve the resolution between the American *Microcyclops* species by considering characters such as the distal region of antennal basis, the maxillary ornamentation, and the thoracic appendages, especially the intercoxal sclerites and medial margin of the basipodite of the first to fourth trunk limbs. Considering a set of 28 morphological characters in adult females, traditional features such as the length ratio of caudal rami, the length: width ratio of the third endopod of the fourth leg, or the length ratios between apical setae of the same segment, appear to be less important for defining differences between very similar species of American *Microcyclops*. In these analyses, the redescription of the Palearctic *M.varicans* was considered, and this species was clearly separated from the American *M.dubitabilis* Kiefer, 1934 and *M.inarmatus* Gutiérrez-Aguirre and Cervantes-Martínez, 2016.

## ﻿Introduction

Deep taxonomic revisions of some freshwater zooplankton Neotropical groups have been carried out in recent decades. These revisions supported that the species richness is still underestimated and the geographic distribution is poorly understood in freshwater zooplankton taxa. For instance, several species considered cosmopolitan, with high phenotypic plasticity and genetic variability, are in fact species complex (usually grouping, five or more species) based upon deep, long-term and wide scale geographical studies (see Kotov et al. 2009; Mercado-Salas et al. 2018; Montoliu-Elena et al. 2019; Mercado-Salas and Suárez-Morales 2021).

Even though a high level of resolution has been reached with some taxonomically problematic groups, incomplete descriptions and lack of designated type (type series) hamper a systematic revision in many Neotropical freshwater zooplankton species, which limits the improvement of the systematic of many taxonomic groups. This kind of taxonomical problem is magnified because of the gaps in knowledge related to taxonomic studies of zooplankton. Either gaps in time, or the interest in faunistic studies are focused on a few groups (Kotov 2016), or because the availability of some techniques, in several regions, is limited.

Some examples of Nearctic, Neotropical, or Pantropical freshwater genera that have recently been reviewed are *Mastigodiaptomus* (Mercado-Salas et al. 2018; Gutiérrez-Aguirre et al. 2020), *Leptodiaptomus* (Silva-Briano and Suárez-Morales 2010), *Mesocyclops* (Hołyńska et al. 2003), *Eucyclops* (Mercado-Salas and Suárez-Morales 2014), *Alona* (Sinev et al. 2005), and *Bosmina* (Kotov et al. 2009). It is notable that in all these cases, new species were described, or new taxonomical arrangements were proposed, based upon a deep taxonomic revision.

In addition, these reviews reveal morphological characters never previously considered or the re-evaluation of refuted characters that facilitate the systematic and faunistic studies of the high diversity in tropical freshwater (Gutiérrez-Aguirre and Cervantes-Martínez 2016). Even with this important progress over the last decades, some genera need further analysis.

In this work, we explore the possibilities of several morphological characters both used and not used in identification keys of *Microcyclops* under the assumption that through classification and ordering models, it is possible to define the species diagnostic characters verifiable by light microscopy observations (in adult females). In addition, the exploration of these characters helped with the redescription of *M.finitimus* Dussart, 1984, and *M.minor* Dussart, 1984, based on type material.

## ﻿Materials and methods

### ﻿Taxonomic analysis

Detailed redescriptions of *Microcyclopsfinitimus* and *M.minor* were based on the morphological and morphometric analyses of adult females recorded as the original material from the type localities. The evaluation included analyses of holotypes deposited in the Copepoda collection of the Muséum national d`Histoire naturelle, Paris (**MNHN**).

### ﻿Data analysis

To normalize the data, meristic magnitudes were square-root transformed and examined to perform two multivariate analyses with the software Multi Variate Statistical Package MVSP 3.1 (Anglesey, UK). A cluster analysis (with UPGMA as a clustering method and Euclidean distance measurement) that grouped specimens with similar morphology and one principal component analysis (PCA) was performed to identify traits that produced the most distinct groups between species (Legendre and Legendre 1998). Only adult females from different populations were considered in both analyses.

The terminology for each appendage follows Huys and Boxshall (1991):

**A1** antennule;

**A2** antenna;

**Md** mandible;

**Mxl** maxillule;

**Mx** maxillae;

**Mxp** maxilliped;

**Bsp** basipodite of swimming legs;

**Enp** endopodal segment;

**Exp** exopodal segment;

**P1–P5** first to fifth swimming legs;

**II** lateral;

**III** outermost;

**IV** outer median;

**V** inner median;

**VI** innermost terminal; and

**VII** dorsal caudal setae.

Biological material deposited in Smithsonian Institution (**USNM**), Staatliches Museum für Naturkunde, Karlsruhe (**SMNK**), Muséum national d’Histoire naturelle, Paris (**MNHN**), and Collection of Zooplankton of El Colegio de la Frontera Sur, Chetumal, Mexico (**ECOCH-CH-Z**) was analyzed. The following morphological characters were considered in the cluster analysis and PCA. When coding was applicable, this is in brackets; abbreviation of each character is presented after a comma. Abbreviations refer to morphological structures listed above:

1. Distal region of antennal basis caudal, A2_DistalCaudal: without spinules on the distal region (1); with spinules on the distal region (2)
2. Distal region of antennal basis frontal, A2_DistalFrontal: without spinules on the distal region (1); with spinules on the distal region (2)
3. Basal seta on maxillary basipodite, MxBsp_BasalSeta: biserially ornamented (1); ornamented on medial margin (2); naked (3)
4. Claw-like projection of maxillary basipodite, MxBsp_Claw: spines arranged on a bump (1); spines arranged on a continuous row (2)
5. Maxillary distal coxal endite, proximal seta, MxEnd_ProxSeta: biserially ornamented (1); one margin ornamented (2)
6. Maxillary distal coxal endite, distal seta, MxEnd_DistSeta: ornamented on medial margin (1); naked (2)
7. Bsp of P1, medial margin of basipodite, BspP1_Medial: with hair-like setae (1); naked (2)
8. Bsp of P1, medial margin, spine ornamentation, BspP1_SpineOrnament: spine with homonomous ornamentation (1); spine with heteronomous ornamentation (2); not apply (3)
9. Second Enp of P1, pores on lateral surface, Enp2P1_pores: without pores (1); one pore (2); two pores (3)
10. The length (L) to width (W) ratio of Enp2P4, Enp2P4_L:W
11. Bsp of P4, medial margin, BspP4_Medial: naked (1); short spine-like setae (2); long hair-like setae (3)
12. The ratio between the lengths of the medial and lateral apical spines of Enp2P4, P4_LMedSpn:LLatSpn
13. The ratio between the lengths of the medial apical spine and Enp2P4, P4_LMedSpn:LEnp2
14. Surface of intercoxal sclerite of P4, P4_IntcxlSclrt: with rows of spinules (1); naked (2); with rows of hair-like setules (3)
15. L to W ratio of the free segment of P5, FSP5_L:W
16. Free segment of P5, medial margin, FSP5_Medial: without spinule (1); with a tiny spinule (2); with a spinule enlarged beyond the apical margin of the free segment (3)
17. The ratio between the lengths of the free segment of the P5 and P5 terminal seta, P5_L-FS:ApclSta
18. L to W ratio of genital double somite, Genital_L:W
19. Presence of spines along the anal somite, Spns_Anal: ventral and dorsal (1); ventral (2)
20. Caudal ramus, the ratio between the lengths of the outer median terminal seta (IV) and outermost terminal seta (III), CR_L-IV:L-III
21. Caudal ramus, the ratio between the lengths of the medial median terminal seta (V) and outermost terminal seta (III), CR_L-V:L-III
22. Caudal ramus, the ratio between the lengths of the innermost terminal seta (VI) and outermost terminal seta (III), CR_L-VI:L-III
23. Caudal ramus, the ratio between the lengths of the innermost terminal seta (VI) and caudal ramus, L-VI:L-CR
24. The ratio between the lengths of the dorsal caudal seta (VII) and caudal ramus, L-VII:L-CR
25. L to W ratio of caudal ramus, CR_L:W
26. Caudal ramus, base of lateral caudal seta (II), CR_Base-II: without spinules (1); with spinules (2)
27. Caudal ramus, the base of outermost terminal seta (III), CR_Base-III: without spinules (1); with spinules (2)
28. Caudal ramus, proportion between point of insertion (measured from anterior of the caudal ramus) of lateral caudal seta (II) and length of lateral margin, Position-II:CR

The sources for the morphological data considered in the multivariate analyses were the type, paratype(s), and other museum specimens (Suppl. material 1). The original descriptions of 11 named species and three named subspecies were also considered, bringing the total to 54 adult females of species recorded in America (Suppl. material 2).

In lack of material, the character states were verified in the original description of the next species: *Microcyclopsvaricans* (G.O. Sars, 1863); *M.ancepspauxensis* (Herbst, 1962); *M.mediasetosus* (Dussart & Frutos, 1985); *M.pumilis* (Pennak & Ward, 1985); and *M.medius* (Dussart & Frutos, 1986).

The matrix showing the distribution of the 28 characters for each species is shown in Table 1. Total likeness between the analyzed specimens was calculated by cluster analyses using Euclidean distance as a similarity index and UPGMA as a linkage method after normalization of data, and performed in a Multi Variate Statistical Package (v. 3.1). After that, principal component analysis (Table 2) was performed to define which of the normalized characters better explain the variability between the species.

**Table 1.** Averages (Av), maximums (Max), and minimums (Min) of the characters analyzed. The abbreviation and state of each character, as noted in Methods.

		*M.pumilis*	*M.ancepspauxensis*	*M.minor*	*M.mediasetosus*	*M.medius*	*M.ceibaensis*	*M.dubitabilis*	*M.inarmatus*	*M.echinatus*	*M.finitimus*	*M.ancepsanceps*	*M.varicans*	*M.elongatus*	*M.furcatus*
A2_DistalCaudal	Av						1	1	1	2	1	1	2		
	Max						1	1	1	2	1	1	2		
	Min						1	1	1	2	1	1	2		
A2_DistalFrontal	Av						1	1	1	1	2	2	1		
	Max						1	1	1	1	2	2	1		
	Min						1	1	1	1	2	2	1		
MxBsp_BasalSeta	Av						3	1	1	2	3	3	2		
	Max						3	1	1	2	3	3	2		
	Min						3	1	1	2	3	3	2		
MxBsp_Claw	Av						2	2	2	2	1	1	2		
	Max						2	2	2	2	1	1	2		
	Min						2	2	2	2	1	1	2		
MxEnd_ProxSeta	Av						2	2	1	1	2	2	2		
	Max						2	2	1	1	2	2	2		
	Min						2	2	1	1	2	2	2		
MxEnd_DistSeta	Av						2	1	1	2	1	1	2		
	Max						2	1	1	2	1	1	2		
	Min						2	1	1	2	1	1	2		
BspP1_Medial	Av		2	1			2	1	1	2	1	1	1	2	
	Max						2	1	1	2	1	1	1		
	Min						2	1	1	2	1	1	1		
BspP1_Spine Ornament	Av	3	3	3	3	1	2	1	2	2	3	3	1	1	1
	Max						2	1	2	2	3	3	1		
	Min						2	1	2	2	3	3	1		
Enp2P1_pores	Av				3		3	1	2	3	2	2	2		
	Max				3		3	1	2	3	2	2	2		
	Min				3		3	1	2	3	2	2	2		
Enp2P4_L:W	Av	2.11	2.71	2.46	2.33	1.83	2.25	1.94	2.18	2.56	2.36	2.52	2.44	2.59	
	Max						2.43	2.18	2.64	2.75	2.5	2.75	2.7		
	Min						2.1	1.75	1.9	2.33	2.22	2.25	2.22		
BspP4_Medial	Av	1	2	3		1	2	2	3	3.2	3	2	2		
	Max						2	2	3	4	3	2	2		
	Min						2	2	3	2	3	2	2		
P4_LMedSpn: LLatSpn	Av	1.37	1.52	1.95	1.22	1.55	1.45	1.90	1.98	2.07	1.39	1.33	1.40	1.75	
	Max						1.7	2.5	2.19	2.26	1.39	1.5	1.50		
	Min						1.45	1.1	1.58	1.9	1.38	1.16	1.25		
P4_LMedSpn: LEnp2	Av	0.57	0.76	0.73	0.76	0.7	0.64	0.85	0.91	0.81	0.78	0.76	0.88	0.49	
	Max						0.74	1.02	0.97	1.02	0.8	0.82	1		
	Min						0.6	0.71	0.86	0.69	0.75	0.7	0.80		
P4_IntcxlSclrt	Av		1	3			1	2	2	1	1	1	2	2	2
	Max						1	2	2	1	1	1	2		
	Min						1	2	2	1	1	1	2		
FSP5_L:W	Av	2.33	3	2	2.66	2.5	3	3.51	3.11	3.77	2.75	2.58	3.6	3.5	2
	Max						3	4.28	4	4	3	2.85	4.2		
	Min						2.6	2.8	2.75	3.66	2.5	2	3.2		
FSP5_Medial	Av	1	2	3	2	1	2	1	2	2	2	3	2	2	1
	Max						2	1	2	2	2	3	2		
	Min						2	1	2	2	2	3	2		
P5_L-FS:ApclSta	Av	0.29	0.18	0.34	0.21	0.33	0.26	0.42	0.28	0.45	0.48	0.41	0.38	0.46	0.40
	Max						0.34	0.58	0.30	0.46	0.53	0.50			
	Min						0.23	0.27	0.26	0.44	0.44	0.23			
Genital_L:W	Av	0.6	1.1	1.06	1.8		0.95	1.02	0.87	1.12	0.94	1.13	1.2		1.41
	Max						1.0	1.2	1	1.22	1.1	1.31			
	Min						0.92	0.9	0.8	1.04	0.78	0.96			
Spns_Anal	Av	1	1	2	1	1	1	1	2	1	1.5	1	2	2	2
	Max						1	1	2	1	2	1	2		
	Min						1	1	2	1	1	1	2		
CR_L-IV:L-III	Av		4.83	6.2	7.3	8.5	5.71	4.59	4.82	6.52	6.05	4.92	5.3	0.54	2.37
	Max						6.24	5.5	5.64	7.26	6.06	5.67	5.4		
	Min						4.67	4.17	3.86	5.52	6.03	4.17	5.2		
CR_L-V:L-III	Av		8.33	8.1	9	13.25	9.95	6.57	7.15	10.48	8.95	7.09	7.14		2.37
	Max						10.4	7.38	7.8	12.52	8.97	8.42	7.14		
	Min						9.63	5.58	6.22	8.54	8.94	6.11	7.14		
CR_L-VI:L-III	Av	0.92	1.81	2.33	3	1	1.82	1.54	1.62	1.96	1.92	1.35	1.64		1
	Max						2.12	1.72	1.88	2.31	2.125	1.74	1.80		
	Min						1.5	1.26	1.29	1.71	1.72	0.96	1.40		
L-VI:L-CR	Av	0.44	1.44	1.16	2.7	0.28	0.81	1.35	1.46	0.56	1.05	0.80	0.93	0.25	0.4
	Max						0.9	1.68	1.51	0.65	1.26	1.08	1		
	Min						0.85	1.1	1.4	0.51	0.85	0.58	0.85		
L-VII:L-CR	Av	0.48	1.55	0.6	0.95	0.58	0.75	1.02	0.89	0.50	0.65	0.55	0.53	0.37	0.2
	Max						1.0	1.22	1.17	0.71	0.78	0.9			
	Min						0.56	0.7	0.71	0.36	0.53	0.4			
CR_L:W	Av	2.9	2.4	3.15	2.29	4.35	3.37	2.51	2.51	5.97	3.40	3.78	3.39	5	6.66
	Max						3.8	3	2.93	6.3	4.1	4.25	3.68		
	Min						3.1	1.88	1.78	5.3	2.7	3	3		
CR_Base-II	Av	1	1	1	2	2	2	1.08	1	2	1	1.08	1	2	1
	Max						2	2	1	2	1	2	1		
	Min						2	1	1	2	1	1	1		
CR_Base-III	Av	1	2	2	2	2	2	2	1	2	2	2	1	1	1
	Max						2	2	1	2	2	2	1		
	Min						2	2	1	2	2	2	1		
Position-II:CR	Av	65.5	68	78	56.25	68.96	69.55	70.12	58.92	73.26	74.70	71.12	68.11	62.5	80
	Max						72.5	76.2	63.15	76.54	75.56	73.33			
	Min						65	61.54	54.00	70.13	73.85	69.12			

**Table 2.** Principal components analysis, variable loadings in bold (analyzing 28 variables for 54 specimens). Data square-root transformed.

	Axis 1	Axis 2	Axis 3
Eigenvalues	1.61	0.46	0.361
Percentage	48.523	13.878	10.886
Cum. Percentage	**48.523**	**62.401**	**73.287**
Genital_L:W	0.078	-0.029	0.011
Enp2P4_L:W	0.074	-0.024	0.06
BspP4_Medial	**0.222**	0.005	-0.14
P4_IntcxlSclrt	0.065	**0.271**	-**0.294**
P4_LMedSpn:LLatSpn	0.059	0.033	-**0.166**
P4_LMedSpn:LEnp2	0.055	0.012	-0.077
CR_L-IV:L-III	**0.25**	-**0.482**	-**0.204**
CR_L-V:L-III	**0.335**	-**0.609**	-**0.237**
CR_L-VI:L-III	0.048	-0.116	-0.093
L-VII:L-CR	0.008	0.01	-**0.184**
L-VI:L-CR	0.022	-0.002	-**0.231**
CR_L:W	0.007	-0.089	**0.275**
CR_Base-II	0.006	-0.1	0.038
CR_Base-III	0.042	-0.104	0.054
Position-II:CR	0.036	-0.071	0.197
Spns_Anal	-0.028	0.054	-0.078
FSP5_L:W	0.038	0.075	-**0.138**
FSP5_Medial	0.064	-0.097	**0.303**
P5_L-FS:ApclSta	0.012	0.02	0.042
BspP1_SpineOrnament	0.024	-**0.174**	**0.35**
BspP1_Medial	**0.16**	0.09	-0.015
MxBsp_BasalSeta	**0.385**	0.097	**0.405**
MxBsp_Claw	**0.311**	**0.261**	-0.278
MxEnd_ProxSeta	**0.318**	**0.26**	0.027
MxEnd_DistSeta	**0.304**	**0.142**	-0.019
A2_DistalCaudal	**0.285**	**0.159**	-0.035
A2_DistalFrontal	**0.284**	**0.153**	0.202
Enp2P1-Pores	**0.339**	-0.05	0.137

## ﻿Results

According to Dussart and Defaye (1995), the morphological characters listed below are diagnostic for *Microcyclops*, and they were present in all the specimens analyzed here:

1. A1 10–12-segmented.
2. Caudal ramus with seta VI as long as, shorter, or longer than seta III.
3. Thoracic limbs (P1–P4) biramous; each ramus 2-segmented. Enp2P4 has two well-developed apical spines, and coxa of P4 has a long-feathered seta on the medial margin.
4. Fifth urosome with fifth leg represented by a lateral seta and a free segment, elongated. The latter bearing one apical seta and with or without one medial spine.

### ﻿Taxonomic account

**Order Cyclopoida Burmeister, 1835**

**Family Cyclopidae Rafinesque, 1815**

**Subfamily Cyclopinae Rafinesque, 1815**

#### Genus *Microcyclops* Claus, 1893

##### ﻿ Microcyclops finitimus

Taxon classificationAnimaliaCyclopoidaCyclopidae

Dussart, 1984

37D3B9F4-F3E4-5829-9823-B8E3173F0912

Figs 1 , 2

Microcyclops finitimus Dussart, 1984: 57, 58, fig. 19A; Dussart 1983: 325, fig. 2B; da Rocha and Por 1998: 2138; da Rocha 1998: 426–431, figs 6, 7, 12, 18; Gutiérrez-Aguirre and Cervantes-Martínez 2016: 54, fig. 14A–E.

###### Material examined.

***Holotype*.** One dissected adult female on a slide labelled as *Microcyclopsfinitimus* female nov. sp. ‘Lagoon’ with *Trapa* between Coporito and Barrancas, Venezuela 24.X.1981, 8h40. Collector Bernard Dussart, and det. B. Dussart (MNHN Cp-678).

###### Other material.

One dissected, adult female on a slide labelled as *Microcyclopsfinitimus* female. Rorota, prés Guyane 21.X.1985. GUYANE. Collector Bernard Dussart, and det. B. Dussart (MNHN Cp-7294).

###### Redescription based on the holotype.

**Figure 1.**
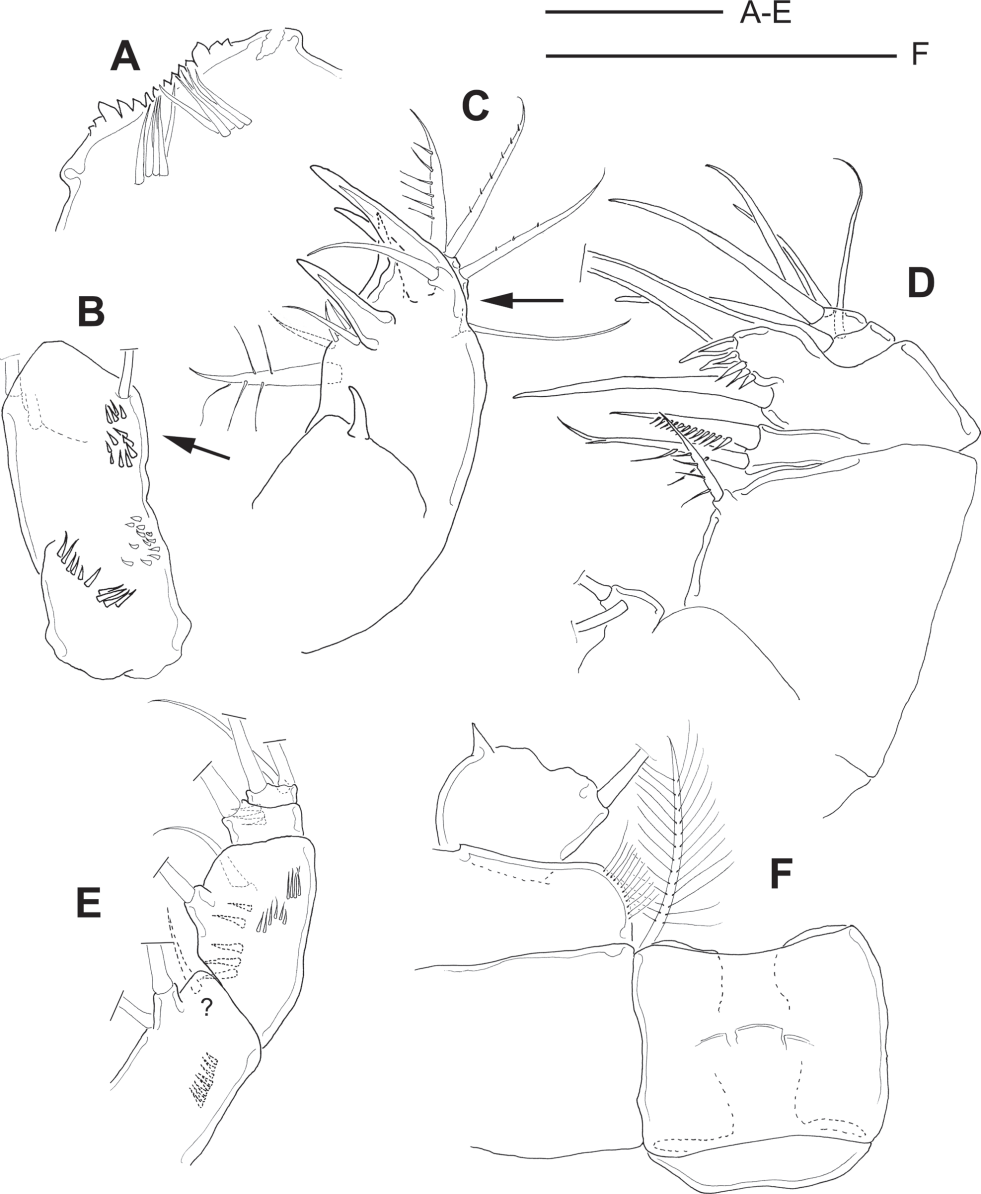
*Microcyclopsfinitimus*. Adult female (MNHN-Cp678) **A** labrum **B** antenna, note that the distal group of spines is arrowed in this caudal-frontal view **C** maxillule, note that the lateral lobe of maxillular palp is missing (area arrowed) **D** maxilla **E** maxilliped, note that the insertion of broken off seta is suggested (indicated by ?) **F** first leg, medial area: intercoxal sclerite, Bsp, and Enp1. Scale bars: 50 µm.

**Female**: body length excluding furcal setae = 0.89 mm (as described by Dussart 1984). Labral plate distally toothed: eight central teeth are flanked by lateral, basally widened teeth, which are followed by two low teeth on each side; medial labral plate with two groups of long, wide setulae; lateral lobes rounded (Fig. 1A).

Antennule 12-segmented: each segment was armed with setae (s), spines (sp) or aesthetascs (ae) in the following order: (1) 8 s; (2) 4 s; (3) 2 s; (4) 6 s; (5) 3 s; (6) 1 s + 1 sp; (7) 2 s; (8) 3 s; (9) 2 s + 1ae; (10) 2 s; (11) 2 s + 1 ae; (12) 7 s + 1 ae.

Antenna with two groups of spinules on the basal margin of the basis in caudal view. In the frontal view antennal basis with two groups of spinules: one next to the exopodal seta, on the distal region (arrowed in Fig. 1B) and one is along the lateral margin.

Maxillule (Fig. 1C): praecoxal arthrite with seven setae. Apical region of maxillary palp with two setae armed with tiny spinules, plus a third seta with long setules. Lateral lobe lost (area arrowed in Fig. 1C). One smooth proximal seta.

Maxillary syncoxal surface smooth (Fig. 1D). Distal coxal endite with two setae: proximal seta distally bifurcated, with long spinules; distal seta with an elongated row of spine-like setules. Basipodite with a bump bearing robust, engrossed spines on the concave margin and one long, bare seta on its base. Enp1 and Enp2 bearing two and three naked and long setae, respectively.

Maxilliped with syncoxa (3 setae, one broken off), basis (2 setae), and two-segmented Enp bearing one and three setae, respectively. Syncoxa, basis, and Enp1 with rows of spinae: basis on frontal and caudal surfaces; syncoxa and Enp1 only on the frontal surface (Fig. 1E).

**Figure 2.**
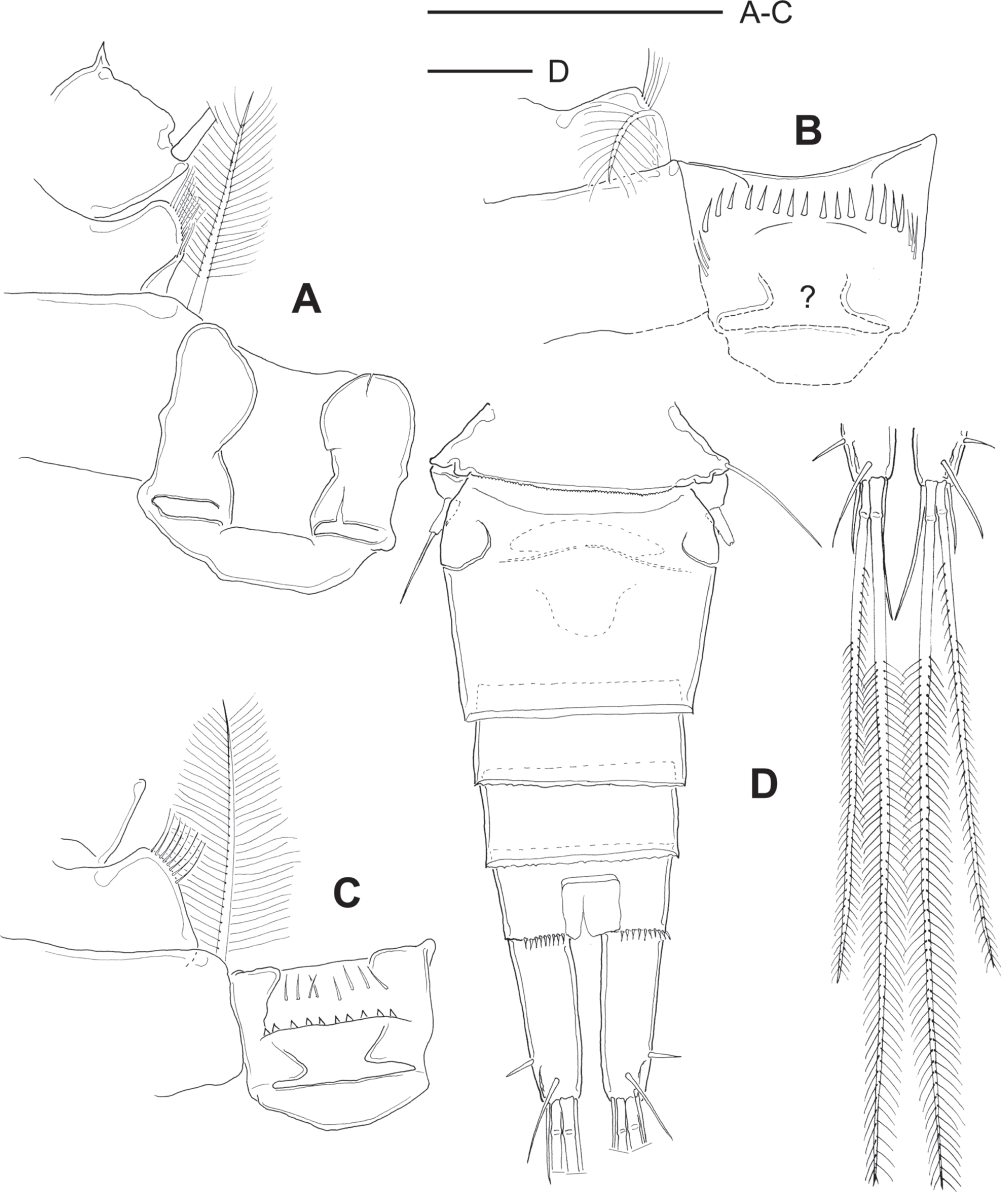
*Microcyclopsfinitimus*. Adult female (MNHN-Cp678) **A** second leg, medial area: intercoxal sclerite, Bsp, and Enp1 **B** third leg, medial area: intercoxal sclerite, and Bsp: the basal area of intercoxal sclerite was not verified (indicated by ?) **C** fourth leg, medial area: intercoxal sclerite, and Bsp**D** urosome, note the separate terminal caudal seta. Scale bars: 50 µm.

Medial margin of basipodites of P1–P4 with long hair-like setae. There is no medial spine on the margin of BspP1 (Fig. 1F). Intercoxal sclerite of P1, and P2 quadrangular, naked (Figs 1F, 2A). Intercoxal sclerite of P3 rectangular with long and robust spinules arranged laterally along the distal margin of the plate (Fig. 2B). Proximal region of the intercoxal sclerite of P3 not observable (indicated by ? in Fig. 2B).

P4 as illustrated and described by Dussart (1984: 57, 58, fig. 19A): intercoxal sclerite rectangular, with two rows of spinules; distal row with elongated spinules, proximal row with short spinules (Fig. 2C). Ratio between the lengths to width of Enp2P4 is 2.2–2.5; the medial spine of Enp2P4 is 1.3× as long as lateral spine and 0.7× as long as the segment.

Fifth pediger bare, with dorsal hyaline membrane serrated posteriorly (Fig. 2D); length to width ratio of genital double somite 0.78. Free segment of P5 3.0× as long as wide, bearing one tiny medial spinule, and 0.4× as long as distal seta. Hyaline fringes of prosomal somites smooth, except the fourth which is serrated; urosomal somites with hyaline fringes slightly serrated. As described by Dussart (1984), length to width ratio of the caudal ramus is 4.1, the inner margin naked; no spinules at the base of the lateral caudal (II) but spines at the base of the outermost terminal (III) caudal setae (spines verified in MHN-Cp7294). Spinae along dorsal and ventral margins of anal somite. Lateral caudal seta (II) inserted 73.0–75.5% of the caudal ramus.

Dorsal caudal seta (VII) 0.5–0.7× as long as caudal ramus, innermost terminal caudal seta (VI) 1.05× as long as caudal ramus. Length ratio between outer median (IV) and outermost terminal seta (III) is 6.0; and between medial median (V) and outermost terminal seta (III) is 8.9 (Fig. 2D).

##### ﻿ Microcyclops minor

Taxon classificationAnimaliaCyclopoidaCyclopidae

Dussart, 1984

023E8E67-ED6A-5DE9-89A2-A262C10968FA

Fig. 3

Microcyclops anceps var. minor Dussart, 1984: 57, fig. 17.

###### Material examined.

***Holotype*.** Dissected, adult female on slide labelled as: Microcyclopsancepsvar.minor [nov. var.]. Charca I, near Unaré river at Clarines (Venezuela), 13.4.1981, Collector Bernard Dussart, and det. B. Dussart (MNHN Cp-673).

###### Redescription based on the holotype.

Dorsal margin of prosomal somites smooth (unfigured). Because of the position of the specimen, it was not possible to observe the buccal appendages.

**Figure 3.**
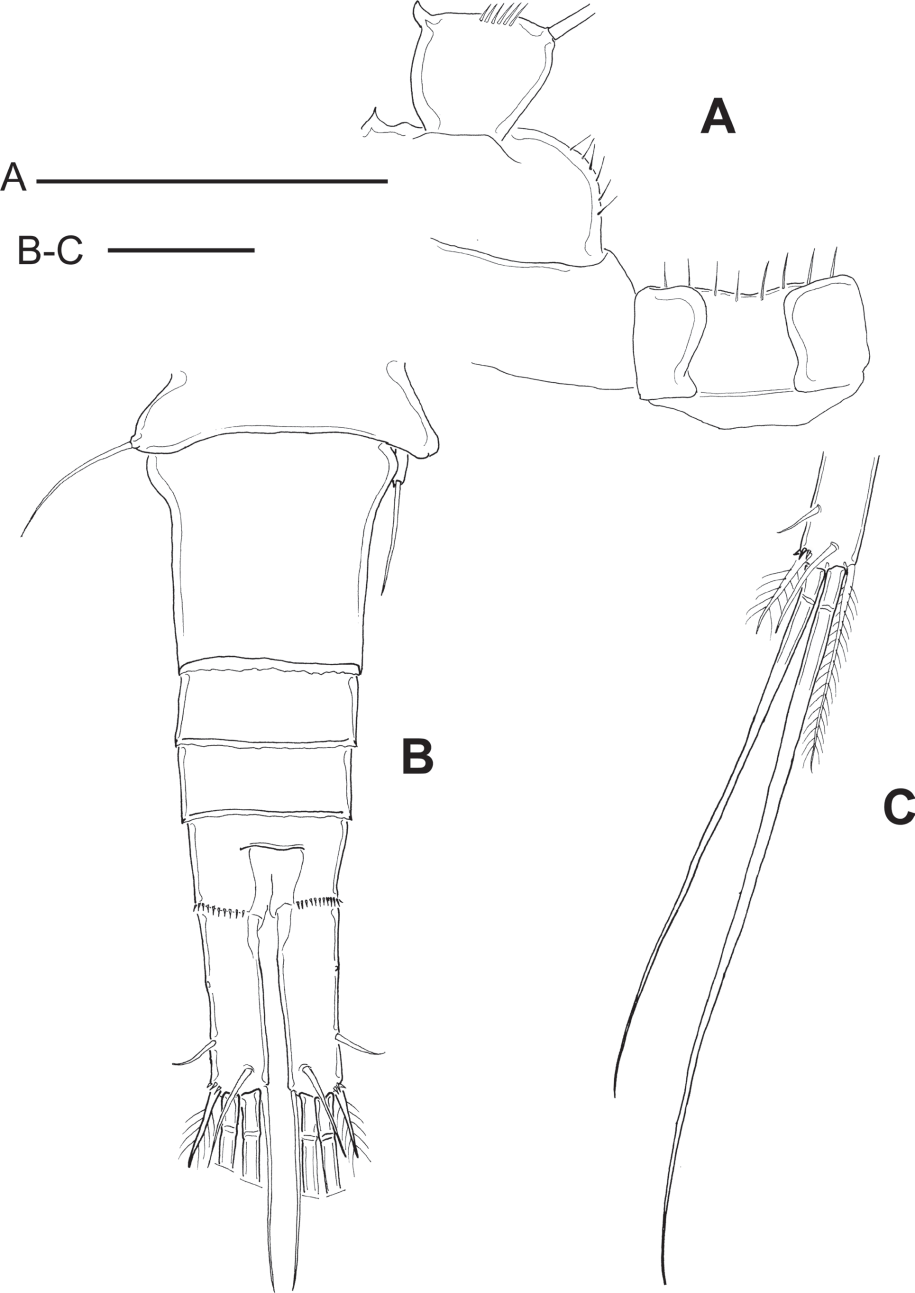
*Microcyclopsminor*. Adult female (MNHN-Cp673) **A** fourth leg, medial area: intercoxal sclerite, Bsp, and Enp1 **B** urosome, dorsal **C** terminal caudal setae. Scale bars 50 µm.

As per the illustration by Dussart (1984), basipodites of P1 with short hair-like setules but without spine on medial margins; coxa with one row of short setules along lateral margins. Basipodite of P4 with short hair-like setules on medial margin (Fig. 3A); P4 intercoxal sclerite rectangular with long setules along distal margin. As illustrated by Dussart (1984) Enp2P4 is 2.46× as long as wide; medial spine 1.95× as long as lateral spine and 0.73× as long as the segment.

Fifth pediger bare, with dorsal hyaline membrane smooth posteriorly; P5 is a cylindrical free segment that bears one apical seta and one projected medial spinule (Fig. 3B). Free segment of P5, 2.0× as long as wide and 0.34× as long as apical seta. Length to width ratio of caudal ramus is 3.15, medial margin naked; no spinules at base of lateral caudal setae, but spinules present at base of outermost terminal caudal setae (Fig. 3B). Short spinules along all the posterior margin of anal somite; lateral caudal seta inserted at 78% of caudal ramus.

Relative lengths of terminal caudal setae from outermost to innermost caudal seta are 1: 6.2: 8.1: 2.33 (Fig. 3C). Due to the differential morphological characteristics previously described, we propose to elevate the status of *M.minor* to the species level.

### ﻿Cluster and PCA analyses

**Figure 4.**
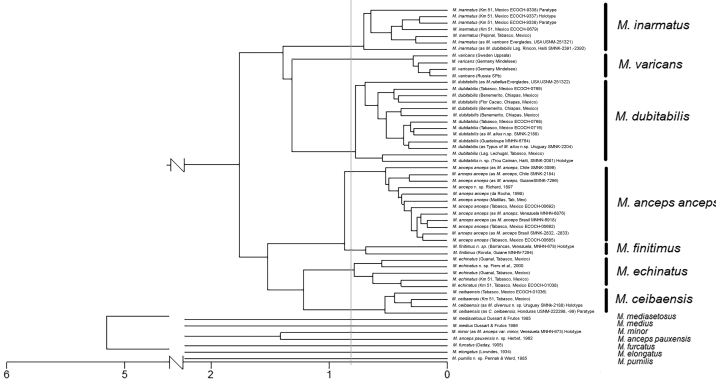
Dendrogram: total morphology likeness between specimens representing the Neotropical species, and *Microcyclopsvaricans*. UPGMA as clustering method and Euclidean distance.

In Fig. 4, the cluster analyses show the grouped specimens according to the total likeness of the 28 considered characters (Table 1). *Microcyclopsmedius* Dussart & Frutos, 1985, *M.mediasetosus* Dussart & Frutos, 1985, *M.minor*, *M.ancepspauxensis* Herbst, 1962, *M.furcatus* (Daday, 1905), *M.elongatus* (Lowndes, 1934), and *M.pumilis* Pennak & Ward, 1985 were observable as isolated entities, because of the lack of information on the buccal appendages in these taxa.

In all species from various geographical regions (Suppl. material 1) *M.inarmatus* Gutiérrez-Aguirre & Cervantes-Martínez, 2016, *M.varicans* (G.O. Sars, 1863), *M.dubitabilis* (Kiefer, 1934), *M.ancepsanceps* (Richard, 1897), *M.finitimus*, *M.echinatus* Fiers, Ghenne & Suárez-Morales, 2000, and *M.ceibaensis* (Marsh, 1919), specimens belonging to the same putative species formed single clusters that were clearly separated from those of other species.

The three groups with the least distance between specimens (the more compact groups in Fig. 4), even though the analyzed specimens were recorded in a wider latitudinal range (Suppl. material 1), were *M.ceibaensis*, *M.varicans*, and *M.ancepsanceps*, but the group with the highest inter- and intrapopulation variability in morphological characters was *M.dubitabilis*. Even though the records are in a more limited latitudinal range (compared to the three previously mentioned species), it is possible to observe discontinuities within the group *M.dubitabilis*.

According to the PCA, all features related to maxilla ornamentation are important characters that explain the model variability in the first axis (Table 2), followed by the presence/absence of cuticular pores on lateral area of Enp2P1, and the ornamentation of the distal region of the antennal basis (with or without a group of spinules on caudal or frontal views). Following the order of importance, the presence or absence of hair-like seta (or another ornament) on the medial margin of BspP4 is also essential, followed by the length ratio between median caudal setae, IV, and V, with the outermost terminal seta (III), and finally, the presence/absence of a hair-like seta on the medial margin of BspP1.

In addition to the characters mentioned before, in Axis 2, the model points that are important characters the ornamentation of the spine on the inner basis of BspP1 (when it is present) and the ornamentation of the intercoxal sclerite of P4 (Table 2).

Axes 1 and 2 together explain 62.4% of the variability, and Axis 3 adds 10% more. In this third axis with values of importance lower than 0.41 (Table 2), the following morphological characters are important according to the model: length ratios between the innermost and dorsal caudal setae with caudal rami length, as well as the length ratio of the medial spine and lateral apical spines of Enp3P4. However, this last character is in a penultimate place (in order of importance), after all other features mentioned before.

## ﻿Discussion

With the analyses performed here, it was clear that the American species *M.inarmatus* and *M.dubitabilis* are not morphologically similar to *M.varicans* (recently redescribed by Mirabdullayev and Defaye 2022), and that the cluster analysis exposes them as different groups.

Some differential morphological characteristics between *M.ancepspauxensis* and *M.minor* had already been previously described in Gutiérrez-Aguirre and Cervantes-Martínez (2016). However, after the analysis of the type material of *M.minor*, the differences between the medial margin of BspP1 and BspP4 are clear, as well as in the ornamentation of the intercoxal sclerite of P4, in the length of the apical seta of P5, and in the length ratio displayed by the terminal furcal setae (see Suppl. material 2). These characters are of importance to delineate between the American species, based on the PCA performed here. Although the evaluation of the buccal appendages of *M.ancepspauxensis* and *M.minor* is still pending, we suggest them as separate species (even distinct from the species *M.ancepsanceps*). To the best of our knowledge, *M.minor* and *M.ancepspauxensis* have only been recorded as original descriptions. Probably the information included herein will encourage the finding of these species in their actual distribution areas.

The majority of *Microcyclops* species occur in the Neotropical region (Suárez-Morales et al. 2020). In addition to the records presented in Suppl. material 1, *Microcyclopsfurcatus* was recorded in São Jose do Norte, Brazil (Cardozo et al. 2007) and Paraguay (Reid 1985). *Microcyclopsfinitimus* and *M.mediasetosus* together with *M.ancepsanceps* and *M.dubitabilis* were recorded in Mato Grosso do Sul, Brazil (da Rocha and Por 1998).

*Microcyclopsancepsanceps* appears to be a Neotropical species with a large geographic range including southeastern Mexico, Guatemala, Venezuela, Guyana, Uruguay, Brazil, and Chile. *Microcyclopsdubitabilis* is also widely distributed in the Neotropics (southeastern Mexico, Florida, Haiti, Guadeloupe, Uruguay, Brazil, and Venezuela).

*Microcyclpsceibaensis* occurs in southeastern Mexico, Central America, and Brazil. *Microcyclopselongatus* was recorded in Brazil and Paraguay, and *M.inarmatus* appears to be distributed in Florida, Haiti, and southeastern Mexico (Gutiérrez- Aguirre and Cervantes-Martínez 2016). To our knowledge, the next species have been recorded exclusively in a single locality: *M.medius* in Argentina (Dussart and Frutos 1986), *M.minor* in Venezuela (Dussart 1984), *M.pumilis* in United States (Pennak and Ward 1985), and *M.ancepspauxensis* in Brazil (Herbst 1962).

*Microcyclopsinarmatus*, *M.varicans*, and *M.dubitabilis* share the character of an armed seta on the maxillary basipodite. In contrast, in *M.ancepsanceps*, *M.finitimus*, *M.echinatus*, and *M.ceibaensis*, the ornamentation of this seta is absent or reduced (in *M.echinatus*). The maxilla has some special features in these four species, such as the row of strong spines on a bump on the concave side of the claw-like seta (in *M.finitimus* or *M.ancepsanceps*); the proximal seta of the maxillary distal coxal endite only ornamented on one side (in *M.ceibaensis*, *M.finitimus*, and *M.ancepsanceps*); the smooth distal seta of the maxillary distal coxal endite (in *M.ceibaensis* and *M.echinatus*). The distribution of these features explains the arrangements in the cluster analysis.

*Microcyclopsfinitimus*, *M.minor*, *M.ancepspauxensis*, *M.pumilis*, *M.mediasetosus*, and *M.ancepsanceps* are the American species that share the absence of spine on the medial margin of the basipodite of first leg. Except for *M.finitimus*, we were not able to observe the buccal structures of most of these species. However, they appear as independent entities (see Fig. 4) because of the clear differences (between species) in the ornamentation of the medial margins of BspP1 and BspP4 (see Table 1), which were of the most important characters to delineate the *Microcyclops* species analyzed here.

Recently, the maxillary and antennal basis microstructure, as well as the structure of swimming legs, especially the ornamentation of intercoxal sclerites and medial margin of basipodite, have been suggested by Einsle (1993), Mirabdullayev (2007), and Gutiérrez-Aguirre and Cervantes-Martínez (2016) as species diagnostic characteristics in *Microcyclops*. Previously da Rocha (1998) pointed out the importance of considering the presence/absence and number of pores on the lateral margin of Enp2P1 as a diagnostic character to recognize Neotropical *Microcyclops* species. The ordination analysis performed here allowed us to distinguish them as the most important characters to be considered in the classification of American *Microcyclops* species.

With this work, it was determined that, indeed, the length ratios (ranges and average) between terminal caudal setae IV:III and V:III are very informative for distinguishing species. These characters are also relatively easy to distinguish using light microscopy; fortunately, they have been illustrated/described in most original descriptions (see Herbst 1962; Dussart 1984) and were observed in the type material examined here.

For the American *Microcyclops* species the statistical analysis also improves the definition that can be achieved in combination with morphological analysis for species resolution, as has been tested with other aquatic species (Lajus et al. 2015; Bradford-Grieve et al. 2017).

Other characteristics that have traditionally been used to differentiate some cyclopoid species are the length ratio of caudal rami, the length ratios in structures on distal endopod of the fourth leg, or the length ratio between dorsal caudal seta and caudal ramus. However, similar to other genera such as *Mesocyclops* or *Eucyclops*, after the analysis surveyed here, these characters can be considered as not informative for differentiating the American *Microcyclops* species because they are shared or have overlapping features (see Table 1).

Additionally, the species *M.echinatus* (in caudal view), *M.finitimus*, *M.ancepsanceps*, and *M.varicans* (in frontal view) share the presence of a group of spines on the distal region of the antennal basis, whereas in *M.inarmatus*, *M.dubitabilis*, and *M.ceibaensis*, this region is bare. The importance of species-specific patterns of teeth and spines on BspA2 has been widely reported in the genera *Macrocyclops*, *Eucyclops*, and *Ectocyclops* in Eucyclopinae (Fiers and Van de Velde 1984; Mercado-Salas and Suárez-Morales 2014) and *Cyclops*, *Mesocyclops*, and *Thermocyclops* in Cyclopinae (Fiers and Van de Velde 1984; Hołyńska 2006; Karanovic et al. 2017). However, for *Microcyclops* species, this pattern has been qualified as remarkably simple, with doubts about its taxonomic value (Fiers and Van de Velde 1984). After this analysis, we confirm that it is possible to differentiate the American *Microcyclops* species when the microstructure (features and position) of teeth and spinules on the caudal or frontal surfaces of A2 are also observed.

## ﻿Conclusions

Two insufficiently known South American species, *M.finitimus* and *M.minor* were redescribed based on type material. According to the classification and ordination models, the microstructure of cephalic appendages, the medial area of thoracic appendages, and the caudal setae of caudal rami (identifiable with light microscopy) were strongly supported as morphological characters that improves resolution between the American *Microcyclops* as well as in species with wide geographic distribution.

## Supplementary Material

XML Treatment for Microcyclops finitimus

XML Treatment for Microcyclops minor

## ﻿Additional information

### Author ORCIDs

Martha Angélica Gutiérrez-Aguirre https://orcid.org/0000-0002-9329-820X

Adrián Cervantes-Martínez https://orcid.org/0000-0002-8947-8558

### Data availability

All of the data that support the findings of this study are available in the main text or Supplementary Information.

## Supplementary materials

Supplementary material 1 Biological material examined Data type Morphological https://binary.pensoft.net/file/887615This dataset is made available under the Open Database License (http://opendatacommons.org/licenses/odbl/1.0/). The Open Database License (ODbL) is a license agreement intended to allow users to freely share, modify, and use this Dataset while maintaining this same freedom for others, provided that the original source and author(s) are credited.Martha Angélica Gutiérrez-Aguirre, Adrián Cervantes-Martínez

Supplementary material 2 Distribution of characters by specimen; abbreviation is explained in data analysis Data type Morphological https://binary.pensoft.net/file/887616This dataset is made available under the Open Database License (http://opendatacommons.org/licenses/odbl/1.0/). The Open Database License (ODbL) is a license agreement intended to allow users to freely share, modify, and use this Dataset while maintaining this same freedom for others, provided that the original source and author(s) are credited.Martha Angélica Gutiérrez-Aguirre, Adrián Cervantes-Martínez
