# Supplementary material for: ﻿Redescription of two species of Microcyclops (Copepoda, Cyclopoida) and use of ordination models to classify American species
Source: Zookeys. 2023 Aug 3;1173:111–30. doi: 10.3897/zookeys.1173.97827 (PMC10416091; doi:10.3897/zookeys.1173.97827)
Supplement: Supplementary material 1 — Biological material examined [file zookeys-1173-111_article-97827__-s001.docx]

Supplementary Table 1. Biological material examined.

| Classified in this study as: | Collection data/Label |
| --- | --- |
| *Microcyclops minor* Dussart, 1984 | Charca I, near Unaré river at Clarines, Venezuela. 06.05.81.  Labelled as: *Microcyclops anceps* var. *minor* [nov. var.]. Holotype MNHN Cp-673 |
| *M. ceibaensis* (Marsh, 1919) | La ceiba Pond, Honduras Acc: 319629 (1 of 1), egg-b, female, ant.abd.ft  Labelled as *Cyclops cubanensis* Marsh number = 4259.  Re-labelled as *Cyclops ceibaensis* Marsh, 1919. Paratype. USNM-222298 |
|  | La ceiba Pond, Honduras Acc: 319629 (1 of 2), ant.antennule.abd.ft,  Labelled as *C. ceibaensis* Marsh, 1919. Paratype. USNM-222299 |
|  | La ceiba Pond, Honduras Acc: 319629 (2 of 2), urosome, maxilla, P4,  Labelled as *C. ceibaensis* Marsh, 1919 Paratype. USNM-222299 |
|  | Two dissected females, Uruguay  Labelled as *Cyclops* (*Microcyclops*) *diversus* n. sp. Kiefer, 1935  SMNK 2188 |
|  | Two dissected females, Uruguay  Labelled as *Cyclops* (*M.*) *diversus* Kiefer, 1935, Abd + P5, 19.11.34  SMNK 2190 |
|  | One dissected female, km 51 Villa hermosa-Frontera, lado 2, Tabasco, Mexico. 13.I.1998  Labelled as *M. ceibaensis* (Marsh, 1919). ECOCH-Z-01036 |
|  | One dissected female, km 51 Villahermosa-Frontera, lado 1, Tabasco, Mexico. 13.I.1998.  Labelled as *M. ceibaensis* (Marsh, 1919). Personal collection |
| *M. dubitabilis* (Kiefer, 1934) | 3 females. Haiti, Trou Caiman (Type locality). 16.02.1933  Labelled as *M. dubitabilis* n. sp. Holotype. SMNK-2081 |
|  | One female, Barra Sta. Luzia, Uruguay; Gr. Teich, 07.01.1933  Labelled as *Cyclops* (*Microcyclops*) *alius* Typus, SMNK-2204 |
|  | One female, Uruguay  Labelled as *Cyclops* (*M.*) *alius* n. sp. SMNK-2189 |
|  | One dissected female, pond with Characeae, Guadeloupe. Labelled as *M. dubitabilis* MNHN-Cp 6764 |
|  | One female, Shark river slough, Everglades, Fla, 1986. Acc: 372909. Col. R. Conrow  Labelled as *Microcyclops rubellus*. USNM-251322 |
|  | One un-dissected female, Charco km 109-198, Flor de Cacao-Benemérito, Chiapas, Mexico,  Labelled as *M. dubitabilis* (Kiefer, 1934). Personal collection |
|  | Two un-dissected and one dissected females, km 154 Benemérito-Palenque, Chiapas, México, 17.04.2000  Labelled as *M. dubitabilis* (Kiefer, 1934). Personal collection |
|  | One un-dissected female, Charco km 20 Jonuta-Villahermosa Tabasco, Mexico. 13.01.1998.  Labelled as *M. dubitabilis* (Kiefer, 1934). ECOCH-0769 |
|  | One un-dissected female, Laguna Lechugal Tabasco, Mexico. 31.I.1999.  Labelled as *M. dubitabilis* (Kiefer, 1934). ECOCH-0708 |
|  | One un-dissected female, Laguna Leona Vicario II, Tabasco, Mexico. 31.I.1999.  Labelled as *M. dubitabilis* (Kiefer,1934). ECOCH-0716 |
|  | One dissected female, Laguna Lechugal Tabasco, Mexico. 31.I.1999.  Labelled as *M. dubitabilis* (Kiefer, 1934). Personal collection |
| *M. inarmatus* Gutiérrez-Aguirre and Cervantes-Martínez, 2016 | One dissected female, km 51 Villahermosa-Frontera, Tabasco lado 1, 13.I.1998. Holotype  Labelled as *M. inarmatus* (in two slides ECOCH-Z-09337). |
|  | Two un-dissected females, km 51 Villahermosa-Frontera, Tabasco lado 1, 13.I.1998. Paratypes  Labelled as *M. inarmatus* ECOCH-Z-09338 |
|  | One dissected female, Laguna Rincon, Haiti. 1.02.1935  Labelled as *M. dubitabilis*. SMNK-2391 |
|  | One dissected female, Laguna Rincon, Haiti. 1.02.1935  Labelled as *M. dubitabilis*. SMNK-2392 |
|  | One dissected female Sites 6, 23, Shark river slough, Everglades National Park, Florida, USA Acc: 372909 (slide 2 of 7). May1986  Labelled as *M. varicans*. USNM-251321 |
|  | One female, Laguna El Pajonal, Tabasco, Mexico. 12.I.1998. Labelled as *M. inarmatus* Gutiérrez-Aguirre and Cervantes-Martínez, 2016. Personal collection |
|  | One un-dissected female, km 51 Villahermosa-Frontera, Tabasco lado 2, 13.I.1998  Labelled as *M. inarmatus* Gutiérrez-Aguirre and Cervantes-Martínez, 2016. ECOCH-Z-0679 |
| *M. echinatus* (Fiers et al., 2000) | One dissected female, km 51 Villahermosa-Frontera, Tabasco, lado 2, 13.I.1998  Labelled as *M. echinatus* ECOCH-Z-01038 |
|  | Two un-dissected females, El Guanal, Litoral II, Tabasco, Mexico, 31.I.1999.  Labelled as *M. echinatus*. Personal collection |
|  | One dissected female (in three slides), km 51 Villahermosa-Frontera, Tabasco lado 1, 13.I.1998.  Labelled as *M. echinatus*. Personal collection |
| *M. finitimus* Dussart, 1984 | One dissected female, Lagoon with *Trapa* between Coporito and Barrancas, Venezuela 24.X.1981, 8h40. Collector Bernard Dussart, and det. B. Dussart. Holotype  Labelled as *M. finitimus* nov. sp. MNHN Cp-678. |
|  | One dissected female, Rorota, prés Guyane 21.X.1985. GUYANE. Collector Bernard Dussart, and det. B. Dussart.  Labelled as *M. finitimus* MNHN Cp-7294. |
| *M. anceps anceps* (Richard, 1897) | One female, Laguna Matillas, Tabasco, Mexico, 12.I.1998 Labelled as *M. anceps anceps* ECOCH-Z-00685 |
|  | Two un-dissected females, Charco 6 Jonuta-Villahermosa, 11.I. 1998  Labelled as *M. anceps anceps* ECOCH-Z-00692 |
|  | One dissected female, Marais Pripriyiyi, Guyane, 23.X.1985  Labelled as *M. anceps anceps* MNHN-Cp7296 |
|  | One dissected female, Uruguay, Abd + P5.  Labelled as *M. anceps* SMNK-2184 |
|  | One dissected female, A1-P4, Maria Farinka, Brasilien, Tümpel, Brazil. 25.08.1935.  Labelled as *M. anceps* SMNK-2832 |
|  | One dissected female, Abd + P5, Maria Farinka, Brasilien, Tümpel. 25.08.1935.  Labelled as *M. anceps* SMNK-2833 |
|  | Un-dissected organisms, 5w + 1m, A-P4, Laguna Larga Magallanes, Chile. 03.02.1931.  Labelled as *M. anceps* SMNK-3099 |
|  | One dissected female, Calabozo reservoir, Venezuela. 15.10.1981  Labelled as *M. anceps* MNHN-Cp6876 |
|  | One un-dissected female, Mare Sans São Carlos (SP) Brazil. 8.04.1982.  Labelled as *M. anceps* MNHN-Cp6918 |
