# Supplementary material for: ﻿Redescription of two species of Microcyclops (Copepoda, Cyclopoida) and use of ordination models to classify American species
Source: Zookeys. 2023 Aug 3;1173:111–30. doi: 10.3897/zookeys.1173.97827 (PMC10416091; doi:10.3897/zookeys.1173.97827)
Supplement: Supplementary material 2 — Distribution of characters by specimen; abbreviation is explained in data analysis [file zookeys-1173-111_article-97827__-s002.docx]

Supplementary Table 2. Distribution of characters by specimen; abbreviation is explained in data analysis.

|  | A2_Distal caudal/frontal | MxBsp_BasalSeta | MxBsp_Claw | MxEnd_ProxSeta | MxEnd_DistSeta | BspP1_Medial | BspP1_SpineOrnament | Enp2P4_L:W | BspP4_Medial | P4_LMedSpn:LLatSpn | P4_LMedSpn:LEnp2 | P4_IntcxlSclrt | FSP5_L:W |
| --- | --- | --- | --- | --- | --- | --- | --- | --- | --- | --- | --- | --- | --- |
| *M. pumilis* Pennak & Ward, 1985 |  |  |  |  |  |  | 3 | 2.11 | 1 | 1.37 | 0.57 |  | 2.33 |
| *M. anceps pauxensis* Herbst, 1962 |  |  |  |  |  | 2 | 3 | 2.71 | 2 | 1.52 | 0.76 | 1 | 3 |
| *M. minor* (MNHN673) holotype |  |  |  |  |  | 1 | 3 | 2.46 | 3 | 1.95 | 0.73 | 3 | 2 |
| *M. mediasetosus* n. sp. Dussart & Frutos, 1985 |  |  |  |  |  |  | 3 | 2.33 |  | 1.22 | 0.76 |  | 2.66 |
| *M. medius* Dussart & Frutos, 1986 |  |  |  |  |  |  | 1 | 1.83 | 1 | 1.55 | 0.7 |  | 2.5 |
| *M. ceibaensis* (USNM-222298, -99) | 1/1 | 3 | 2 | 2 | 2 | 2 | 2 | 2.1 | 2 | 1.67 | 0.6 | 1 | 3 |
| *M. ceibaensis* (as *M. diversus* SMNK-2188, -2190) | 1/1 | 3 | 2 | 2 | 2 | 2 | 2 | 2.43 | 2 | 1.53 | 0.6 | 1 | 2.7 |
| *M. ceibaensis* (ECOCHZ-01036) | 1/1 | 3 | 2 | 2 | 2 | 2 | 2 | 2.29 | 2 | 1.7 | 0.74 | 1 | 2.6 |
| *M. ceibaensis* (km51 Vill-Frntra1) | 1/1 | 3 | 2 | 2 | 2 | 2 | 2 | 2.25 | 2 | 1.45 | 0.64 | 1 | 3 |
| *M. dubitabilis* (SMNK-2081) holotype | 1/1 | 1 | 2 | 2 | 1 | 1 | 1 | 1.75 | 2 | 2.5 | 0.91 | 2 | 3.6 |
| *M. dubitabilis* (as *M. alius* SMNK-2204) | 1/1 | 1 | 2 | 2 | 1 | 1 | 1 | 1.8 | 2 | 1.61 | 0.75 | 2 | 3 |
| *M. dubitabilis* (as *M. alius* SMNK-2189) | 1/1 | 1 | 2 | 2 | 1 | 1 | 1 | 2.1 | 2 | 1.86 | 1.02 | 2 | 2.8 |
| *M. dubitabilis* (MNHN-6764) | 1/1 | 1 | 2 | 2 | 1 | 1 | 1 | 1.87 | 2 | 1.91 | 0.95 | 2 | 2.8 |
| *M. dubitabilis* (as *M. rubellus* USNM-251322) | 1/1 | 1 | 2 | 2 | 1 | 1 | 1 | 2.1 | 2 | 1.1 | 0.8 | 2 | 4 |
| *M. dubitabilis* (Flor Cacao, Chiapas, Mex) | 1/1 | 1 | 2 | 2 | 1 | 1 | 1 | 2.18 | 2 | 2.15 | 0.8 | 2 | 4 |
| *M. dubitabilis* (Benemerito, Chiapas, Mex) | 1/1 | 1 | 2 | 2 | 1 | 1 | 1 | 1.97 | 2 | 2.12 | 0.86 | 2 | 4.28 |
| *M. dubitabilis* (Benemerito, Chiapas, Mex) | 1/1 | 1 | 2 | 2 | 1 | 1 | 1 | 1.75 | 2 | 1.92 | 0.77 | 2 | 3.25 |
| *M. dubitabilis* (Benemerito, Chiapas, Mex) | 1/1 | 1 | 2 | 2 | 1 | 1 | 1 | 2.17 | 2 | 2.24 | 0.97 | 2 | 4 |
| *M. dubitabilis* (ECOCHZ-0769) | 1/1 | 1 | 2 | 2 | 1 | 1 | 1 | 1.75 | 2 | 1.66 | 0.71 | 2 | 4.28 |
| *M. dubitabilis* (ECOCHZ-0708) | 1/1 | 1 | 2 | 2 | 1 | 1 | 1 | 1.8 | 2 | 1.6 | 0.85 | 2 | 3 |
| *M. dubitabilis* (ECOCHZ-0716) | 1/1 | 1 | 2 | 2 | 1 | 1 | 1 | 2.05 | 2 | 2 | 0.84 | 2 | 2.8 |
| *M. dubitabilis* (Lag. Lechugal) | 1/1 | 1 | 2 | 2 | 1 | 1 | 1 | 1.97 | 2 | 2 | 0.85 | 2 | 3.85 |
| *M. inarmatus* (as *M. dubitabilis* SMNK-2391, -2392) | 1/1 | 1 | 2 | 1 | 1 | 1 | 2 | 1.9 | 3 | 1.58 | 0.97 | 2 | 2.85 |
| *M. inarmatus* (as *M. varicans* USNM-251321) | 1/1 | 1 | 2 | 1 | 1 | 1 | 2 | 2.64 | 3 | 1.9 | 0.86 | 2 | 3 |
| *M. inarmatus* (Pajonal, Tabasco, Mex) | 1/1 | 1 | 2 | 1 | 1 | 1 | 2 | 2.27 | 3 | 2.19 | 0.9 | 2 | 3 |
| *M. inarmatus* (ECOCHZ-0679) | 1/1 | 1 | 2 | 1 | 1 | 1 | 2 | 2.05 | 3 | 2.18 | 0.89 | 2 | 4 |
| *M. inarmatus* (km51, lado2) | 1/1 | 1 | 2 | 1 | 1 | 1 | 2 | 2.19 | 3 | 1.97 | 0.91 | 2 | 2.75 |
| *M. inarmatus* (km51, lado2) | 1/1 | 1 | 2 | 1 | 1 | 1 | 2 | 2 | 3 | 2.09 | 0.92 | 2 | 2.95 |
| *M. inarmatus* (km51, lado2) | 1/1 | 1 | 2 | 1 | 1 | 1 | 2 | 2.19 | 3 | 1.97 | 0.91 | 2 | 3.23 |
| *M. echinatus* (ECOCHZ-01038) | 2/1 | 2 | 2 | 1 | 2 | 2 | 2 | 2.75 | 4 | 2.04 | 0.81 | 1 | 3.7 |
| *M. echinatus* (Guanal, Tab, Mex) | 2/1 | 2 | 2 | 1 | 2 | 2 | 2 | 2.68 | 4 | 2.26 | 0.79 | 1 | 3.66 |
| *M. echinatus* (Km51 Vill, Tab, Mex) | 2/1 | 2 | 2 | 1 | 2 | 2 | 2 | 2.33 | 4 | 2 | 1.02 | 1 | 4 |
| *M. echinatus* (Fiers et al., 2000) | 2/1 | 2 | 2 | 1 | 2 | 2 | 2 | 2.6 | 2 | 2.14 | 0.76 | 1 | 3.7 |
| *M. echinatus* (Guanal, Tab, Mex) | 2/1 | 2 | 2 | 1 | 2 | 2 | 2 | 2.44 | 2 | 1.9 | 0.69 | 1 | 3.8 |
| *M. finitimus* (MNHN7294) | 1/2 | 3 | 1 | 2 | 1 | 1 | 3 | 2.22 | 3 | 1.39 | 0.8 | 1 | 2.5 |
| *M. finitimus* (MNHN678) holotype | 1/2 | 3 | 1 | 2 | 1 | 1 | 3 | 2.5 | 3 | 1.38 | 0.75 | 1 | 3 |
| *M. anceps anceps* (ECOCHZ-00685) | 1/2 | 3 | 1 | 2 | 1 | 1 | 3 | 2.75 | 2 | 1.27 | 0.76 | 1 | 2.5 |
| *M. anceps anceps* (Matillas, Tab, Mex) | 1/2 | 3 | 1 | 2 | 1 | 1 | 3 | 2.54 | 2 | 1.39 | 0.82 | 1 | 2.8 |
| *M. anceps anceps* (ECOCHZ-00692) | 1/2 | 3 | 1 | 2 | 1 | 1 | 3 | 2.72 | 2 | 1.37 | 0.73 | 1 | 2.53 |
| *M. anceps anceps* (ECOCHZ-00692) | 1/2 | 3 | 1 | 2 | 1 | 1 | 3 | 2.6 | 2 | 1.35 | 0.76 | 1 | 2.85 |
| *M. anceps anceps* (as *M. anceps* MNHN-7296) | 1/2 | 3 | 1 | 2 | 1 | 1 | 3 | 2.53 | 2 | 1.5 | 0.72 | 1 | 2.6 |
| *M. anceps anceps* (as *M. anceps* SMNK-2184) | 1/2 | 3 | 1 | 2 | 1 | 1 | 3 | 2.25 | 2 | 1.26 | 0.76 | 1 | 2.7 |
| *M. anceps anceps* (as *M. anceps* SMNK-2832, 2833) | 1/2 | 3 | 1 | 2 | 1 | 1 | 3 | 2.4 | 2 | 1.3 | 0.73 | 1 | 2.5 |
| *M. anceps anceps* (as *M. anceps* SMNK-3099) | 1/2 | 3 | 1 | 2 | 1 | 1 | 3 | 2.47 | 2 | 1.16 | 0.78 | 1 | 2.6 |
| *M. anceps ancep*s (as *M. anceps* MNHN-6876) | 1/2 | 3 | 1 | 2 | 1 | 1 | 3 | 2.5 | 2 | 1.47 | 0.73 | 1 | 2.8 |
| *M. anceps anceps* (as *M. anceps* MNHN-6918) | 1/2 | 3 | 1 | 2 | 1 | 1 | 3 | 2.4 | 2 | 1.3 | 0.7 | 1 | 2.6 |
| *M. anceps* Richard, 1897 | 1/2 | 3 | 1 | 2 | 1 | 1 | 3 | 2.53 | 2 | 1.2 | 0.8 | 1 | 2 |
| *M. anceps anceps* (da Rocha, 1998) | 1/2 | 3 | 1 | 2 | 1 | 1 | 3 | 2.5 | 2 | 1.4 | 0.8 | 1 | 2.5 |
| *M. varicans* (Russia) | 2/1 | 2 | 2 | 2 | 2 | 1 | 1 | 2.3 | 2 | 1.2 | 1 | 2 | 3.2 |
| *M.* *varicans* (Sweden) | 2/1 | 2 | 2 | 2 | 2 | 1 | 1 | 2.4 | 2 | 1.3 | 0.8 | 2 | 4.2 |
| *M. varicans* (Germany) | 2/1 | 2 | 2 | 2 | 2 | 1 | 1 | 2.2 | 2 | 1.3 | 0.8 | 2 | 3.3 |
| *M. varicans* (Germany) | 2/1 | 2 | 2 | 2 | 2 | 1 | 1 | 2.6 | 2 | 1.4 | 0.9 | 2 | 3.8 |
| *M. elongatus* (Lowndes, 1934) |  |  |  |  |  | 2 | 1 | 2.59 |  | 0.49 | 1.75 | 2 | 3.5 |
| *M. furcatus* (Daday, 1905) |  |  |  |  |  |  | 1 |  |  |  |  | 2 | 2 |

Supplementary Table 2. Continuation

|  | FSP5_Medial | P5_L-FS:ApclSta | Genital_L:W | Spns_Anal | CR_L-IV:L-III | CR_L-V:L-III | CR_L-VI:L-III | L-VI:L-CR | L-VII:L-CR | CR_L:W | CR_Base-II | CR_Base-III | Position-II:CR |
| --- | --- | --- | --- | --- | --- | --- | --- | --- | --- | --- | --- | --- | --- |
| *M. pumilis* Pennak & Ward, 1985 | 1 | 0.29 | 0.6 | 1 |  |  | 0.92 | 0.44 | 0.48 | 2.9 | 1 | 1 | 65.5 |
| *M. anceps pauxensis* Herbst, 1962 | 2 | 0.18 | 1.1 | 1 | 4.83 | 8.33 | 1.81 | 1.44 | 1.55 | 2.4 | 1 | 2 | 68 |
| *M. minor* (MNHN673) holotype | 3 | 0.34 | 1.06 | 2 | 6.2 | 8.1 | 2.33 | 1.16 | 0.6 | 3.15 | 1 | 2 | 78 |
| *M. mediasetosus* Dussart & Frutos, 1985 | 2 | 0.21 | 1.8 | 1 | 7.3 | 9 | 3 | 2.7 | 0.95 | 2.29 | 2 | 2 | 56.25 |
| *M. medius* Dussart & Frutos, 1986 | 1 | 0.33 |  | 1 | 8.5 | 13.25 | 1 | 0.28 | 0.58 | 4.35 | 2 | 2 | 68.96 |
| *M. ceibaensis* (USNM-222298, -99) | 2 | 0.25 | 0.94 | 1 | 5.89 | 10 | 1.77 | 0.65 | 1 | 3.8 | 2 | 2 | 65 |
| *M. ceibaensis* (as *M. diversus* SMNK-2188, -2190) | 2 | 0.23 | 1 | 1 | 6.05 | 9.63 | 1.89 | 0.9 | 0.82 | 3.1 | 2 | 2 | 69.57 |
| *M. ceibaensis* (ECOCHZ-01036) | 2 | 0.34 | 0.97 | 1 | 4.67 | 9.8 | 1.5 | 0.87 | 0.62 | 3.47 | 2 | 2 | 71.15 |
| *M. ceibaensis* (km51 Vill-Frntra1) | 2 | 0.25 | 0.92 | 1 | 6.24 | 10.4 | 2.12 | 0.85 | 0.56 | 3.13 | 2 | 2 | 72.58 |
| *M. dubitabilis* (SMNK-2081) holotype | 1 | 0.58 | 0.9 | 1 | 5 | 7 | 1.72 | 1.15 | 0.7 | 2.7 | 1 | 2 | 61.54 |
| *M. dubitabilis* (as *M. alius* SMNK-2204) | 1 | 0.5 | 1.2 | 1 | 4.6 | 6.56 | 1.26 | 1.1 | 1.04 | 2.3 | 1 | 2 | 75 |
| *M. dubitabilis* (as *M. alius* SMNK-2189) | 1 | 0.53 | 1.2 | 1 | 4.54 | 6.7 | 1.55 | 1.11 | 1.03 | 2.38 | 1 | 2 | 71 |
| *M. dubitabilis* (MNHN-6764) | 1 | 0.5 | 0.96 | 1 | 4.4 | 6.67 | 1.47 | 1.47 | 1.2 | 1.88 | 1 | 2 | 75 |
| *M. dubitabilis* (as *M. rubellus* USNM-251322) | 1 | 0.27 | 0.9 | 1 | 5.5 | 7.38 | 1.34 | 1.13 | 0.92 | 2.6 | 1 | 2 | 76.2 |
| *M. dubitabilis* (Flor Cacao, Chiapas, Mex) | 1 | 0.4 | 1 | 1 | 4.35 | 6.5 | 1.64 | 1.55 | 1.22 | 2.46 | 1 | 2 | 63.51 |
| *M. dubitabilis* (Benemerito, Chiapas, Mex) | 1 | 0.3 | 1 | 1 | 4.17 | 6.6 | 1.39 | 1.52 | 1.21 | 2.35 | 1 | 2 | 75 |
| *M. dubitabilis* (Benemerito, Chiapas, Mex) | 1 | 0.3 | 1 | 1 | 4.17 | 5.58 | 1.64 | 1.65 | 1.06 | 2.5 | 1 | 2 | 67.05 |
| *M. dubitabilis* (Benemerito, Chiapas, Mex) | 1 | 0.5 | 1 | 1 | 4.17 | 5.76 | 1.68 | 1.68 | 1.17 | 2.42 | 1 | 2 | 70.58 |
| *M. dubitabilis* (ECOCHZ-0769) | 1 | 0.58 | 0.96 | 1 | 4.5 | 6.5 | 1.67 | 1.43 | 1.06 | 2.92 | 1 | 2 | 68.57 |
| *M. dubitabilis* (ECOCHZ-0708) | 1 | 0.4 | 0.92 | 1 | 4.79 | 6.64 | 1.43 | 1.14 | 0.8 | 2.69 | 1 | 2 | 71.43 |
| *M. dubitabilis* (ECOCHZ-0716) | 1 | 0.3 | 1.10 | 1 | 4.55 | 6.52 | 1.61 | 1.47 | 1 | 2.4 | 1 | 2 | 72.22 |
| *M. dubitabilis* (Lag. Lechugal) | 1 | 0.34 | 1.09 | 1 | 4.92 | 7 | 1.69 | 1.22 | 0.81 | 3 | 2 | 2 | 64.44 |
| *M. inarmatus* (as *M. dubitabilis* SMNK-2391, -2392) | 2 | 0.26 | 0.8 | 2 | 5.64 | 7.8 | 1.29 | 1.4 | 1.17 | 1.78 | 1 | 1 | 54 |
| *M. inarmatus* (as *M. varicans* USNM-251321) | 2 | 0.28 | 0.9 | 2 | 5.5 | 7.7 | 1.5 | 1.43 | 0.93 | 2.65 | 1 | 1 | 63.15 |
| *M. inarmatus* (Pajonal, Tabasco, Mex) | 2 | 0.3 | 0.81 | 2 | 4.97 | 7.6 | 1.88 | 1.51 | 0.81 | 2.65 | 1 | 1 | 62.26 |
| *M. inarmatus* (ECOCHZ-0679) | 2 | 0.28 | 0.84 | 2 | 3.86 | 7 | 1.70 | 1.43 | 0.80 | 2.93 | 1 | 1 | 56.82 |
| *M. inarmatus* (km51, lado2) | 2 | 0.27 | 0.87 | 2 | 4.72 | 6.48 | 1.70 | 1.5 | 0.9 | 2.56 | 1 | 1 | 58 |
| *M. inarmatus* (km51, lado2) | 2 |  | 1 | 2 | 4.70 | 6.22 | 1.70 | 1.5 | 0.71 | 2.47 | 1 | 1 | 59.52 |
| *M. inarmatus* (km51, lado2) | 2 | 0.28 | 0.87 | 2 | 4.34 | 7.28 | 1.59 | 1.44 | 0.91 | 2.54 | 1 | 1 | 58.67 |
| *M. echinatus* (ECOCHZ-01038) | 2 | 0.45 | 1.04 | 1 | 5.81 | 8.54 | 1.81 | 0.51 | 0.71 | 6.09 | 2 | 2 | 74.35 |
| *M. echinatus* (Guanal, Tab, Mex) | 2 | 0.44 | 1.22 | 1 | 7 | 11.75 | 2.15 | 0.53 | 0.41 | 6.23 | 2 | 2 | 76.54 |
| *M. echinatus* (Km51 Vill, Tab, Mex) | 2 | 0.46 | 1.09 | 1 | 5.52 | 9.6 | 1.84 | 0.60 | 0.52 | 5.92 | 2 | 2 | 70.13 |
| *M. echinatus* (Fiers et al., 2000) | 2 | 0.45 | 1.14 | 1 | 7 | 10 | 1.71 | 0.65 | 0.53 | 5.3 | 2 | 2 | 70.9 |
| *M. echinatus* (Guanal, Tab, Mex) | 2 | 0.46 | 1.1 | 1 | 7.26 | 12.52 | 2.31 | 0.53 | 0.36 | 6.3 | 2 | 2 | 74.39 |
| *M. finitimus* (MNHN7294) | 2 | 0.53 | 1.1 | 1 | 6.06 | 8.94 | 2.13 | 1.26 | 0.78 | 2.7 | 1 | 2 | 75.56 |
| *M. finitimus* (MNHN678) holotype | 2 | 0.44 | 0.78 | 2 | 6.03 | 8.97 | 1.72 | 0.85 | 0.53 | 4.1 | 1 | 2 | 73.85 |
| *M. anceps anceps* (ECOCHZ-00685) | 3 | 0.41 | 1.16 | 1 | 4.57 | 7.43 | 1.23 | 0.67 | 0.45 | 4 | 1 | 2 | 70.31 |
| *M. anceps anceps* (Matillas, Tab, Mex) | 3 | 0.33 | 0.96 | 1 | 4.17 | 6.11 | 1.31 | 0.76 | 0.56 | 3.44 | 1 | 2 | 72.58 |
| *M. anceps anceps* (ECOCHZ-00692) | 3 | 0.41 | 1.12 | 1 | 5 | 6.64 | 1.33 | 0.79 | 0.43 | 4.13 | 1 | 2 | 71.21 |
| *M. anceps anceps* (ECOCHZ-00692) | 3 | 0.33 | 1.31 | 1 | 4.58 | 6.5 | 1.5 | 0.88 | 0.51 | 4.25 | 1 | 2 | 69.12 |
| *M. anceps anceps* (as *M. anceps* MNHN7296) | 3 | 0.4 | 1.1 | 1 | 5.53 | 8.42 | 1.74 | 1.03 | 0.4 | 3.56 | 1 | 2 | 70.3 |
| *M. anceps anceps* (as *M. anceps* SMNK-2184) | 3 | 0.5 | 0.98 | 1 | 4.5 | 7.4 | 1.2 | 0.7 | 0.5 | 4 | 1 | 2 | 71 |
| *M. anceps anceps* (as *M. anceps* SMNK-2832, 2833) | 3 | 0.5 | 1.09 | 1 | 5.67 | 7.67 | 1.73 | 1.08 | 0.67 | 3 | 1 | 2 | 72 |
| *M. anceps anceps* (as *M. anceps* SMNK-3099) | 3 | 0.45 | 1.2 | 1 | 5.3 | 7.2 | 1.3 | 0.8 | 0.6 | 4.1 | 2 | 2 | 71.8 |
| *M. anceps ancep*s (as *M. anceps* MNHN-6876) | 3 | 0.48 | 1.3 | 1 | 5 | 7.1 | 1.39 | 0.78 | 0.5 | 3.5 | 1 | 2 | 70.5 |
| *M. anceps anceps* (as *M. anceps* MNHN-7296) | 3 | 0.48 | 1.1 | 1 | 5.2 | 6.6 | 1.3 | 0.7 | 0.5 | 3.8 | 1 | 2 | 71 |
| *M. anceps* Richard, 1897 | 3 | 0.23 | 1.125 | 1 | 4.43 | 6.78 | 0.96 | 0.58 | 0.53 | 3.8 | 1 | 2 | 70.27 |
| *M. anceps anceps* (da Rocha, 1998) | 3 | 0.45 | 1.08 | 1 | 5.09 | 7.27 | 1.18 | 0.87 | 0.9 | 3.75 | 1 | 2 | 73.33 |
| *M. varicans* (Russia) | 2 | 0.38 | 1.2 | 2 | 5.3 | 7.14 | 1.6 | 0.96 | 0.53 | 3.5 | 1 | 1 | 68.1 |
| *M.* *varicans* (Sweden) | 2 | 0.37 | 1.1 | 2 | 5.4 | 7.1 | 1.4 | 0.85 | 0.5 | 3.68 | 1 | 1 | 68 |
| *M. varicans* (Germany) | 2 | 0.35 | 1.2 | 2 | 5.2 | 7.14 | 1.7 | 0.93 | 0.52 | 3.2 | 1 | 1 | 68.1 |
| *M. varicans* (Germany) | 2 | 0.38 | 1.0 | 2 | 5.3 | 7.1 | 1.87 | 1 | 0.53 | 3.48 | 1 | 1 | 68 |
| *M. elongatus* (Lowndes, 1934) | 2 | 0.46 |  | 2 |  |  | 0.54 | 0.25 | 0.37 | 5 | 2 | 1 | 62.5 |
| *M. furcatus* (Daday, 1905) | 1 | 0.4 | 1.41 | 2 | 2.37 | 2.37 | 1 | 0.4 | 0.2 | 6.66 | 1 | 1\| | 80 |
